# SOCIOECONOMIC STATUS AND INTERGENERATIONAL LIVING ARRANGEMENTS: BOTH CHILD- AND PARENTS-BASED ANALYSES

**DOI:** 10.1093/geroni/igae098.2734

**Published:** 2024-12-31

**Authors:** Mary Beth Ofstedal, BoRin Kim, Jersey Liang, Xiao Xu, James Raymo

**Affiliations:** University of Michigan, Ann Arbor, Michigan, United States; University of New Hampshire, Durham, New Hampshire, United States; University of Michigan, Ann Arbor, Michigan, United States; Columbia University, New York, New York, United States; Princeton University, Princeton, New Jersey, United States

## Abstract

Intergenerational living arrangements are closely associated with intra-family transfers of support and decision-making between older parents and adult children. Guided by the rational choice and the life course frameworks, we simultaneously examined the linkages between socioeconomic status of older parents and their adult children and changes in their living arrangements over a 20-year period. Data came from the 1998-2018 Health and Retirement Study. Our sample was restricted to parents over age 65 (n=6,284) and their children over age 21 (n=31,289) at the baseline. The number of total person-period observations over 20 years was 152,344. Three-level hierarchical linear models were used to examine the association of parents’ and children’s socioeconomic characteristics with their living arrangements, including co-, proximate (≤10miles), and distant residence (>10miles). The most common living arrangement for children over time was distant residence, followed by proximate residence. During the study period, the predicted probability of coresidence increased from 1% to 4%, whereas the probability of distant residence declined from 72% to 64%. Proximate residence remained relatively stable at 27% to 32%. The socioeconomic status of both generations was important. Low income and assets of parents and/or children increased the probability of coresidence and decreased the probability of proximate residence. Higher education of parents and children was associated with distance residence. Interestingly, parents’ health status was not significantly associated with living arrangements. Results highlight the importance of accounting for the opportunities, resources and preferences of both older parents and their children when examining intergenerational living arrangements.

